# Understanding essential aspects of fibromyalgia care through a qualitative study of patient feedback

**DOI:** 10.1016/j.pecinn.2026.100466

**Published:** 2026-03-06

**Authors:** Catherine Pressimone, Caroline Cao, Jillian Kyle, Rachel Vanderberg

**Affiliations:** a Department of Medicine, University of Pittsburgh Medical Center (UPMC), Pittsburgh, USA; b Department of Medicine, University of Pittsburgh School of Medicine, Pittsburgh, USA

**Keywords:** Fibromyalgia, Chronic pain, Patient experience, Provider communication, Qualitative study, patient-centered care, empathy

## Abstract

**Objectives:**

Fibromyalgia (FM) is a common, chronic pain syndrome; however, patients with FM report that providers are often ill-equipped to manage this condition. We created an internal medicine-based FM Clinic to address this void and provide evidence-based, patient-centered FM care. This qualitative study aimed to analyze patient experience in the FM Clinic.

**Methods:**

Patients were eligible for this study if they met the American College of Rheumatology 2016 diagnostic criteria for FM, attended at least 2 clinic appointments, and completed at least two Revised Fibromyalgia Impact Questionnaires (FIQRs). Eligible patients were interviewed regarding overall experience, helpful clinic features, and areas for improvement. Results were qualitatively analyzed to identify themes.

**Results:**

Fifty-six patients met inclusion criteria and were interviewed. Four overarching themes were identified: (1) prior negative healthcare experiences, (2) appreciation for safe spaces, (3) identification of clinic features that optimized FM care, and (4) areas for improvement.

**Innovation:**

Current patient perspectives regarding FM care delivery models are limited and focused on non-pharmacologic group-based interventions. This study is the first to explore patient perspectives on an individualized care delivery model.

**Conclusions:**

This study identifies patient-valued elements of FM care that can inform the development of similar care models or FM care provided in other clinical settings.

## Highlights

• Patients with fibromyalgia reported prior negative health care experiences.
• Participants felt a dedicated fibromyalgia clinic provided a safe space.
• Empathy, validation, and listening were key to creating a sense of safety.
• Positive clinic features included individualized care and support/education.
• Participants reported a desire for ongoing, longitudinal care and support.

## 1 Background

Fibromyalgia (FM) is a common, complex, chronic pain condition affecting an estimated 2–5% of the population [1], [2], [3], [4]. FM is characterized by diffuse, widespread body pain accompanied by sleep disturbances, non-restorative sleep, and cognitive fog with many patients also experiencing headaches, mood disturbance, and gastrointestinal upset [1], [3], [5], [6]. Despite ongoing advancements in biomarker research [7], FM remains a clinical diagnosis, defined by established diagnostic criteria centered on widespread pain and symptom burden [8], [9]. The etiology of FM is not fully understood and remains an area of active research [7]. Current treatment guidelines recommend a multimodal approach including nonpharmacologic therapies such as graded exercise, pharmacologic therapies, and behavioral health interventions [6], [10], [11].

There are several challenges in FM care including low provider knowledge, inadequate provider communication skills, stigma or misconceptions about FM, and lack of a clearly defined medical home [3], [5], [12], [13], [14], [15], [16]. FM care remains fragmented among multiple specialties including primary care providers, rheumatologists, and/or pain management specialists [17], [18]. Patients report frequent negative experiences with the healthcare system including a lengthy diagnostic process, poor care coordination and continuity, limited support, inadequate appointment time, and a focus on medications rather than a holistic approach [17]. Patients with FM often feel illegitimized and invalidated when interacting with the healthcare system [17], [19], [20]. The inadequacy of care, especially patient-provider communication, has been linked with several negative consequences including decreased patient satisfaction, decreased adherence with treatment modalities, and worse clinical outcomes [21], [22]. The perceived difficulty in management can also lead providers to feel that caring for patients with FM is difficult, frustrating, and unrewarding [13].

To address the existing gap in FM care within our healthcare system, we created an internal medicine-based FM Clinic that delivers evidence-based, interdisciplinary, patient-centered care. The aim of this study was to analyze patient experience to iteratively refine and improve the FM Clinic. Given the novel nature of the clinic, we used an exploratory qualitative approach without preconceptions.

## 2 Methods

### 2.1 Fibromyalgia clinic structure

The FM Clinic was created via an institutional collaboration between the divisions of Rheumatology and General Internal Medicine at The University of Pittsburgh Medical Center located in Pittsburgh, Pennsylvania. The FM Clinic receives referrals from rheumatology after initial evaluation excludes autoimmune disease. The FM Clinic consists of two physicians, two pharmacists, one licensed professional counselor, and nursing support. The clinic offers evidenced-based pharmacologic management options including selective serotonin re-uptake inhibitors, tricyclic antidepressants, gabapentinoids, muscle relaxers, low-dose naltrexone, and medical cannabis [5], [6], [10], [11], [23], [24]. Non-pharmacologic interventions include referrals to the licensed professional counselor for non-manualized, supportive cognitive behavioral therapy, physical therapy, aqua therapy, and chronic pain occupational therapy [25], [26], [27], [28]. The FM clinic provides individual care over approximately six months consisting of one pharmacist visit, four physician visits and additional visits for nonpharmacologic therapy as needed with the intention of transitioning care to the patient's primary care provider once symptoms are improved or have reached a manageable level.

### 2.2 Data collection

The electronic medical record (EMR) was queried to identify all patients seen in the FM clinic between February 2021 and June 2022 (*n* = 146). On retrospective chart review 56 patients met inclusion criteria: fulfilled American College of Rheumatology 2016 diagnostic criteria for FM, attended a minimum of 2 FM Clinic appointments, graduated from the FM Clinic, and completed at least two Revised Fibromyalgia Impact Questionnaires (FIQRs) (one during the initial visit and one at a subsequent visit) [8], [29]. The FIQR was routinely administered as an objective marker of disease severity [29]. The qualitative survey included three questions about overall experience, helpful aspects of the clinic, and aspects of the clinic that could be improved. Patient responses were collected via written responses through EMR messaging and individual phone interviews. EMR messages were sent by an administrative assistant, and phone interviews were conducted by an internal medicine resident (CP); neither were involved in the FM Clinic. Phone interviews were conducted between November 2022 and March 2023 and transcribed verbatim.

### 2.3 Data analysis

We used the Standards for Reporting Qualitative Research (SRQR) guidelines (Supplement 1) and NVivo coding software to assist in our analysis [30] of both written feedback and phone transcripts. We developed a qualitative codebook using an editing or inductive style in which researchers develop codes from ideas or concepts identified within the transcripts [31]. Two authors unaffiliated with the FM clinic, CC and CP, performed iterative analyses. First, they independently coded two thirds of the transcripts then met to adjudicate differences and developed a final code book before re-coding the entire text with final adjudication to 100% agreement. All data was coded and included in the final analysis; however, CC and CP noted that thematic saturation was reached after coding approximately two thirds of the transcripts. We used two coders as a method of triangulation through multiple analysts to bolster the credibility of our findings [32]. Coding was examined for patterns in participant responses to identify overarching themes. We assessed face validity of identified themes by discussing our findings with others engaged in the care of patients with FM including other members of our clinic team and attendees at national conferences [32].

### 2.4 Ethics

This study was approved by the University of Pittsburgh Institutional Quality Improvement Committee (ID: 4026, approved 10/10/2022). Patients received a standardized script describing the purpose and voluntary nature of the project and provided verbal informed consent to participate. Written documentation of consent was waived given the minimal-risk quality improvement designation. This research did not receive grant funding.

## 3 Results

Fifty-six patients met inclusion criteria and all 56 were interviewed. Participants' demographics are presented in Table 1.

**Table 1:** Participant demographics (*N* = 56).

Characteristic	Value
Age, mean (years)	45
Female sex, n (%)	50/56 (86%)
Non-Hispanic White, n (%)	51 (91%)
Baseline FIQR^a^ score, mean	61^b^

a Fibromyalgia Revised Index Questionnaire.

b moderate disease severity [40].

Four overarching themes were identified: (1) prior negative healthcare experiences, (2) appreciation for safe spaces, (3) identification of clinic features that optimized FM care and (4) areas for improvement. Table 2 presents supporting quotations for each theme.

**Table 2:** Thematic analysis supporting quotations regarding patient experience in a fibromyalgia clinic.

Theme	Subtheme	Patient feedback
Prior negative healthcare experiences		“I thought for 15 years that I had fibromyalgia and I couldn't get anyone to listen to me…. So many people told me I was just fat. It took me so long and I suffered.” (Patient 1) “No one else had answers for me or tried to understand me.” (Patient 2) “It took me absolutely many, many, many years to find someone to identify fibromyalgia as a real syndrome.” (Patient 3) “Since I was diagnosed it's felt like a joke to people, even other providers.” (Patient 4) “A lot of fibro warriors don't even have providers that believe it's a real thing.” (Patient 5) “Sometimes I forget that it is there. Not because I don't have symptoms but because other providers ignore it.” (Patient 6) “Every time I go to someone else, like my PCP [primary care physician], they never know what to say.” (Patient 7) “This wasn't someone telling me it was all in my head or made up – which has happened to me.” (Patient 8) “I was going to like three appointments a week before [FM Clinic] trying to figure out what was wrong with me.” (Patient 10)
Fibromyalgia clinic as a safe space		“Really welcoming and everyone was really nice.” (Patient 9) “She was the first doctor I had seen in a really long time that listened to me.” (Patient 10) “I love that they listened. They listened well and to everything that I said. And didn't treat me like I was crazy.” (Patient 11) “I feel like I'm being heard.” (Patient 12) “Listened to me, really listened to me and took me seriously.” (Patient 1) “They listen and they understand. You finally acknowledged it.” (Patient 13) “I feel heard here.” (Patient 2) “Takes her time with you, she has open communication, and really answers your questions.” (Patient 14) “The clinic, just having a doctor that asks about it, accepts it as a real syndrome is a huge asset.” (Patient 3) “I really like the ability to talk about my symptoms and have them believe me and sympathize with me.” (Patient 15) “My problem was recognized as existent… It was very validating.” (Patient 6) “I liked how friendly and open everyone was. No one made me feel like I was crazy.” (Patient 24)
Aspects of the clinic which optimized patient care	Accessibility	“I could choose to be seen over a virtual [appointment] or in person… Her willingness to discuss things over messaging worked better for me too.” (Patient 16) “The virtual options were helpful while I was working.” (Patient 17) “I also appreciate that I can use the [patient messaging] site to communicate instead of playing phone tag.” (Patient 18)
	Multimodal care/alternative care options	“The OT was very helpful, probably number 1.” (Patient 19) “I also love that she helped me do medical marijuana to help me avoid pills.” (Patient 1) “Helped me get my medical marijuana card.” (Patient 20) “Very helpful with medication.” (Patient 21) “Increased my meds, and I'm feeling much better now.” (Patient 22)
	Individualized care	“Has my best interests in mind.” (Patient 23) “Really liked the ‘where do you want to be’ question and then the goal was to get me there.” (Patient 24) “I love that she tested me for my vitamins as soon as I asked it too. If she hadn't listened to me, we would have missed it. She was just really there for me, and it was personalized care.” (Patient 11) “I appreciated how much work [the providers] did to meet my needs.” (Patient 16) “… provided me with real options - my pain levels are at the best they've been at in years.” (Patient 18)
	Support	“[Clinic providers] really [help] me communicate to my other providers.” (Patient 24) “My back is covered now so if I need anything I know who to call.” (Patient 2) “I finally didn't feel nuts anymore. The clinic is the only place I get that, and it's the only place that will give me answers to specific fibromyalgia questions.” (Patient 15) “…so thankful to have this resource.” (patient 5)
	Patient education	“They were able to give me insight about fibromyalgia.” (Patient 25) “Helped me to understand fibro better.” (Patient 21) “I didn't realize the way I was moving was adding to my pain.” (Patient 19) “I learned that some of the things I didn't even attribute to my fibromyalgia diagnosis were perfectly explained by it.” (Patient 15) “Having a specialist focus on it helped me remember how much it impacts the way other health issues affect me.” (Patient 6)
Areas for improvement		“It would be nice to be seen again on even a less regular schedule because it's designed to treat temporarily and then pass off to a PCP, but my PCP isn't really helping me with my fibromyalgia.” (Patient 26) “Allow/enable more/longer term follow-up. My PCP wouldn't prescribe or continue my meds (pregabalin) and said he didn't feel comfortable helping me with it. Annual follow-up would be great.” (Patient 27)

The first theme emphasized prior negative health care experiences. Participants reported they were not taken seriously, stigmatized, or readily dismissed at other healthcare visits. Participants highlighted that these negative experiences led to personal hardships including delays in diagnosis and high healthcare utilization.

The second theme focused on the sense of safety provided by the FM Clinic. Key elements to creating a safe space included expressing sympathy or empathy, active listening, and validation. Several participants commented on the positive impact of feeling heard and noted that this was the first time their symptoms were viewed as legitimate.

In the third theme, participants highlighted several beneficial clinic features including accessibility, multimodal or alternative care options, individualized care, support, and education. Participants appreciated the accessibility of virtual visits and inter-visit communication through the EMR messenger. Participants noted the benefits of non-pharmacologic therapies such as occupational therapy and nontraditional treatments such as medical cannabis. Further, participants appreciated the individualized care provided through goal setting and holistic care. FM education and ongoing support/resources were also important features for participants.

Areas for improvement that participants identified included physical clinic modifications such as providing coffee in the waiting room and easier parking to help minimize fatigue. Many individuals expressed a desire for long-term follow-up with the FM Clinic.

## 4 Discussion and conclusion

### 4.1 Discussion

This study suggests that the FM clinic was generally well received by patients. The findings reinforce prior literature demonstrating frequent negative healthcare encounters for patients with FM, the importance of patient-centered communication, and key elements of chronic pain management such education, multimodal treatment, and longitudinal care [14], [15], [17], [19], [20], [33], [34], [35], [36], [37], [38], [39]. Important components of care not previously identified [35], [36], [37], [38], [39] include the convenience of video visits and EMR messaging and the willingness of physicians to discuss nontraditional treatments. Patient desire to involve family members to enhance education and understanding has been noted in other studies, but not in this current study [35], [36], [37].

One strength of this study is that all data was coded to 100% agreement by two coders to enhance validity of the findings. Limitations include single institution design, small sample size, and study population homogeneity. Female sex predominance may reflect disease epidemiology. We tried to mitigate social desirability bias by using interviewers unaffiliated with the FM Clinic; however, this may still be a limitation given the vulnerability of the patient population.

### 4.2 Innovation

Research on FM care has largely focused on treatment approaches, with less attention on structured care delivery models [11], [17]. Other FM clinics or programs are largely group-based programs with brief (typically 12 weeks or less) but intensive (8–150 h of contact) interventions [34], [35], [36], [37], [38], [39]. In contrast, the FM clinic emphasizes individualized care over a longer period with variable intensity depending upon patients' participation in nonpharmacologic modalities. The literature examining patient perspective regarding FM care delivery models is limited and is focused on non-pharmacologic group-based interventions [35], [36], [37], [38], [39]. Group program benefits include peer support, sharing similar experiences, and expanding social networks, but many participants still requested individualized treatment [35], [36], [37], [38], [39]. This study expands the literature by exploring patient perspectives on an individualized care delivery model. The FM clinic could likely benefit from a group component, but the current findings highlight that patients do value individualized FM care.

### 4.3 Conclusion

This study identifies patient-valued elements of FM care that can inform the development of similar care models or FM care provided in other clinical settings.

## CRediT authorship contribution statement

**Catherine Pressimone:** Writing – review & editing, Formal analysis, Data curation. **Caroline Cao:** Writing – original draft, Formal analysis. **Jillian Kyle:** Writing – review & editing, Supervision, Methodology, Conceptualization. **Rachel Vanderberg:** Writing – review & editing, Supervision, Software, Resources, Methodology, Conceptualization.

## Declaration of competing interest

The authors have no disclosures or conflicts of interest.
